# Characterizing the Dysfunctional NK Cell: Assessing the Clinical Relevance of Exhaustion, Anergy, and Senescence

**DOI:** 10.3389/fcimb.2020.00049

**Published:** 2020-02-13

**Authors:** Sean J. Judge, William J. Murphy, Robert J. Canter

**Affiliations:** ^1^Division of Surgical Oncology, Department of Surgery, University of California, Davis, Sacramento, CA, United States; ^2^Department of Dermatology, University of California, Davis, Sacramento, CA, United States; ^3^Department of Medicine, University of California, Davis, Sacramento, CA, United States

**Keywords:** natural killer cells, NK cells, NK dysfunction, NK exhaustion, immune dysfunction

## Abstract

There is a growing body of literature demonstrating the importance of T cell exhaustion in regulating and shaping immune responses to pathogens and cancer. Simultaneously, the parallel development of therapeutic antibodies targeting inhibitory molecules associated with immune exhaustion (such as PD-1, but also TIGIT, and LAG-3) has led to a revolution in oncology with dramatic benefits in a growing list of solid and hematologic malignancies. Given this success in reinvigorating exhausted T cells and the related anti-tumor effects, there are increasing efforts to apply immune checkpoint blockade to other exhausted immune cells beyond T cells. One approach involves the reinvigoration of “exhausted” NK cells, a non-T, non-B lymphoid cell of the innate immune system. However, in contrast to the more well-defined and established molecular, genetic, and immunophenotypic characteristics of T cell exhaustion, a consensus on the defining functional and phenotypic features of NK “exhaustion” is less clear. As is well-known from T cell biology, separate and distinct molecular and cellular processes including senescence, anergy and exhaustion can lead to diminished immune effector function with different implications for immune regulation and recovery. For NK cells, it is unclear if exhaustion, anergy, and senescence entail separate and distinct entities of dysfunction, though all are typically characterized by decreased effector function or proliferation. In this review, we seek to define these distinct spheres of NK cell dysfunction, analyzing how they have been shown to impact NK biology and clinical applications, and ultimately highlight key characteristics in NK cell function, particularly in relation to the role of “exhaustion.”

## Introduction

Reinvigorating exhausted cytotoxic CD8 T cells through checkpoint blockade therapy targeting PD-1 and PD-L1 has led to dramatic benefits in a growing list of solid and hematologic malignancies (Wei et al., 2018). While the understanding of T cell exhaustion has greatly expanded since initial ground-breaking publications by Moskophidis et al. (1993) and Gallimore et al. (1998), critical features have remained constant: (1) exhaustion occurs through persistent antigen exposure which interferes with standard immune contraction mechanisms and classic T cell memory formation, and (2) subsequent effector function is diminished, seemingly because of the development of the exhausted state (Wherry, 2011; Wherry and Kurachi, 2015). In contrast to this prototypical model of T cell exhaustion (which is critically dependent on chronic and persistent antigen exposure), it is important to acknowledge that other relevant forms of T cell dysfunction exist, namely anergy and senescence, and in these states, different stimuli and pathways are involved producing different manifestations of dysfunction with variable reversibility.

Given this background, the last several years have witnessed significant interest in applying checkpoint blockade therapy to natural killer (NK) cells, primarily to exploit their well-known anti-tumor functions and similarities to CD8 T cells (Narni-Mancinelli et al., 2011; Sun and Lanier, 2011). Yet, while NK and CD8 T cells share many effector traits, the activation, inhibition, and generation of effector functions in NK cells is distinct from cytotoxic T cells, with complex mechanisms involved. Although there are many similarities between cytotoxic T cells and NK cells, leading many to hypothesize that NK cells could be targeted for reversal of “exhaustion” because of these similarities, the certainty of this approach remains undefined. In this review, we summarize the evidence for NK exhaustion as well as other states of dysfunction (including anergy and senescence) and discuss how these dysfunctional states are similar and different, highlighting how critical differences exist between NK and T cell dysfunctional states with implications for clinical application.

## NK Cells and Anti-Tumor Effects

Since the initial descriptions of NK cell activity nearly 50 years ago (Cudkowicz and Stimpfling, 1964; Cudkowicz and Bennett, 1971) and subsequent identification of a distinct NK lymphocyte population (Kiessling et al., 1975), NK cells have been pursued as an anti-tumor therapy due to their ability to kill transformed cells in an MHC-unrestricted manner. This defining characteristic of NK cells contrasts with the antigen specificity of cytotoxic CD8 T cells, which mediate MHC-restricted killing following antigen presentation and T cell priming. Additionally, loss of MHC-I expression occurs in multiple malignancies and has been associated with T cell immune evasion (Garrido et al., 1976, 2016; Restifo et al., 1993; Algarra et al., 1997; Garcia-Lora et al., 2001; Carretero et al., 2008). However, MHC-I downregulation has also been shown to activate NK cells via the “missing-self” hypothesis (Kärre, 2008). These features of NK targeting further support the concept of using NK cells therapeutically to augment tumor killing, especially since T cells are often rendered ineffective by cancer immunoediting, antigen loss variants, and MHC-I downregulation (Mittal et al., 2014). However, despite this paradigm that MHC-I positive tumor cells can be targeted by cytotoxic T cells and MHC-I negative tumor cells can be eliminated by NK cells, the clinical benefit of NK cell-based therapy has overall been modest, especially for solid tumors, and true breakthrough successes have been limited (Suen et al., 2018; Miller and Lanier, 2019).

Clinical trials using NK cells for the treatment of hematologic and solid malignancies have been ongoing for decades (Suen et al., 2018; Hu et al., 2019; Miller and Lanier, 2019). More recent efforts have focused on the *ex vivo* activation and expansion of peripheral NK cells using antigen presenting cells transfected with co-stimulatory ligands and membrane-bound cytokines to achieve high numbers of cells for adoptive therapy (Fujisaki et al., 2009b; Somanchi et al., 2011; Denman et al., 2012). These approaches were developed with the idea that NK cells produced in this way are highly functional, and greater numbers are needed *in vivo* to obtain a measurable anti-tumor effect. In addition to the massive expansion using these feeder-line approaches, these *ex vivo* NK cells are highly activated as shown by cytotoxicity assays against a range of tumor cell lines *in vitro* (Fujisaki et al., 2009b; Garg et al., 2012). However, despite impressive *in vitro* data on NK cytotoxicity using feeder-line expansion, expanded NK cells using these techniques tend to lose function quickly *in vivo* post-adoptive transfer, consistent with the relatively disappointing results of clinical trials irrespective of whether autologous or allogeneic NKs are used (Suen et al., 2018). Results like these have prompted interest in testing other NK sources, such as *in vitro* activated and expanded NK cell lines (e.g., NK-92) as a lower cost, consistent source of allogeneic cells which may overcome barriers to maintaining activation of NK cells following transfer *in vivo*. However, despite similarly impressive pre-clinical data showing high cytotoxicity and significant anti-tumor effects *in vitro* (Klingemann et al., 2016; Suck et al., 2016), results from clinical trials using NK-92 cells have also been modest (Arai et al., 2008; Tonn et al., 2013). Importantly, these discrepancies between *in vitro* and *in vivo* function of NK cells highlight several key observations which likely underlie their unrealized/disappointing clinical potential, namely that continuous stimulation with cytokines, or target cell activation results in acute increases in effector function but at the same time creating a state of cytokine/activating-signal dependence which then leads to rapid loss of function and survival if these activating/stimulatory signals are taken away. These fundamental observations about NK cell dysfunction post-adoptive transfer have led to intense investigation into strategies to reverse NK dysfunction *in vivo* by several different mechanisms, including overexpression of co-stimulatory molecules, pharmacologic doses of stimulatory cytokines, and combination with checkpoint blockade inhibitors (Miller and Lanier, 2019). As use of checkpoint blockade therapy is ubiquitous and increasingly being applied to NK-based therapy, a critical assessment of the extent and mechanisms of NK dysfunction, including exhaustion, is warranted. Techniques utilized in the expansion and activation of NK cells (i.e., cytokines, feeder line co-culture, co-stimulatory molecules) may give rise to heightened activation, but also dysfunction, and further may lead to NK cells “addicted” to supraphysiologic stimulatory signals that can never be safely reproduced in a human recipient following adoptive cell transfer. These dysfunction pathways likely impact the success (or failure) of NK-based clinical trials, and a better understanding of the spectrum of NK dysfunction pathways will allow for improved clinical application of NK cells, including how and when NK cells might respond to checkpoint blockade therapy to reverse NK “exhaustion.”

## Defining NK Cell Dysfunction

**Figure 1:**
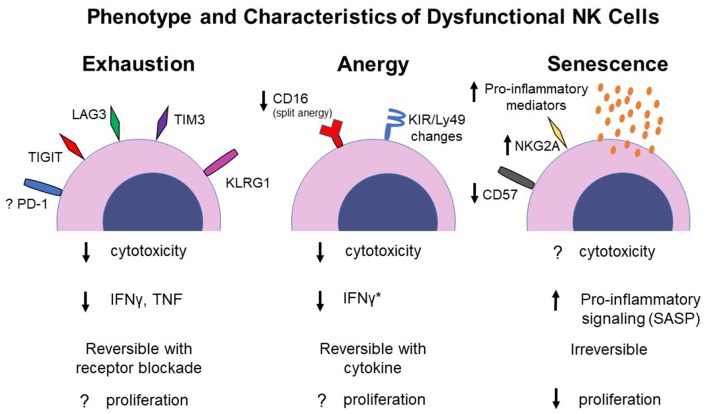
Proposed phenotype and functional changes of NK cells under different dysfunctional states. Specific pro-inflammatory mediators associated with SASP in NK cells have not been determined. *IFNγ production has been shown to increase in split anergized NK cells. SASP, senescence-associated secretory phenotype.

Dysfunctional NK cells are frequently identified by decreased expression of typical NK effector functions in a NK population of interest (such as tumor-infiltrating NK cells) compared to those of a control population (such as circulating NK cells in the peripheral blood) from the same host (Carrega et al., 2008; Carlsten et al., 2009). In general, readouts for NK effector function include *in vitro* cytotoxicity assays against target cells as well as IFNγ and granzyme B production. As these characteristics are generic markers of a dysfunctional NK cell, different states of NK dysfunction, such as anergy and exhaustion, become blurred because there is no established NK phenotype for these dysfunctional states (Figure 1). A further challenge in defining the etiology and spectrum of NK cell dysfunction lies in the fact that a thorough understanding of NK ontogeny and maturation remain ill-defined in many cases. And although there are increasing articles and reviews investigating NK cell exhaustion, there remains controversy as to whether NK cells even undergo exhaustion (as opposed to other dysfunction processes), and if so, the phenotypic markers that define an exhausted NK cell. This is in contrast to T cell development and maturation states which are well-characterized and annotated (Crotty and Rafi, 2004; Koch and Radtke, 2011). These knowledge gaps in some of the basic understanding of NK cell biology (Caligiuri, 2008) add difficulty to defining how, why and when NK cell dysfunction occurs.

Induced T cell dysfunction by either exhaustion, anergy or senescence is believed (in most cases) to protect the host from adverse autoimmune disease or immunopathology (Schwartz, 2003; Blank et al., 2019). This reinforces the well-known principle in immunology that the host must balance the anti-microbial/anti-tumor effects of immune defense with the risks of immunopathology from an unrestrained immune response. As virtually all human autoimmune disease is either B- or T-cell mediated (Davidson and Betty, 2001; Marrack et al., 2001; Rosenblum et al., 2015), the need for induced NK cell dysfunction to limit autoimmune disease is much less clear, with conflicting evidence suggesting both helpful and harmful roles of NK cells in autoimmunity (Schleinitz et al., 2010). Distinct from autoimmunity, there does seem to be toxicities associated with highly activated NK cells as recently described following a clinical trial of adoptively transferred *ex vivo* activated NK cells (Shah et al., 2015), suggesting that mechanisms for activation-induced NK dysfunction may be beneficial in regards to limiting host toxicities.

## NK Cell Exhaustion

Identification and reversal of NK “exhaustion” is a current active field of investigation at both the basic and translational level (Table 1). In contrast to seminal studies on T cell exhaustion which have focused on viral models and human patients with chronic viral infections, studies investigating NK cell exhaustion have focused primarily on identifying dysfunctional tumoral-associated NK cells and applying strategies to reverse NK exhaustion to augment anti-tumor effects (da Silva et al., 2014; Beldi-Ferchiou et al., 2016; Seo et al., 2017; Zhang et al., 2018). However, the contribution of NK cells to the tumor immune infiltrate is generally considered to be low (Whiteside and Parmiani, 1994; Halama et al., 2011; Melero et al., 2014), and the clinical relevance of intra-tumoral NK cells remains incompletely characterized (Pagès et al., 2010), highlighting the need for a more thorough understanding of how NK cells home to diverse solid tumors. In the study from Halama et al., the authors set out to compare the NK and T cell infiltrate in colorectal cancer specimens (Halama et al., 2011). Using NKp46 to detect NK cells via IHC, the authors showed that NK cells were substantial in normal colonic mucosa, but limited within adjacent adenomas or carcinomas. A similar trend was detected in liver metastases, which had significantly less infiltrating NK cells that adjacent normal liver. Furthermore, the limited NK infiltrate detected had no correlation with tumor cell HLA class I expression, suggesting that other mechanisms contribute to the paucity of NK cell infiltration (Halama et al., 2011).

**Table 1:** Summary of key studies examining NK cell exhaustion marker expression in different contexts and populations.

**References**	**Species**	**Population**	**Exhaustion marker(s)**	**Key findings and significance**	
Benson et al. (2010)	Human	Multiple Myeloma	PD-1	HD: 1.4% ± 0.35 MM: 64% ± 4.4	^a^First paper to show NK PD-1.
Wiesmayr et al. (2012)	Human	PTLD	PD-1	HD: 14% ± 6 PTLD: 36% ± 24	Variable expression of PD-1 on NK cells in healthy and PTLD patients.
da Silva et al. (2014)	Human	Melanoma	Tim-3 PD-1	Tim-3 HD: ~20-90% MD: ~25-90% PD-1 HD: <2% MD: <2%	NK PD-1 absent in healthy and melanoma patients. Proposes role for Tim-3 in mediating NK exhaustion in advanced melanoma.
MacFarlane et al. (2014)	Human	RCC	PD-1	MFI: RCC>HD in CD56^dim^ subset	Links NK PD-1 and disease stage, but low PD-1 MFI overall, and differences only compared to healthy donors.
Beldi-Ferchiou et al. (2016)	Human	Kaposi's Sarcoma	PD-1	HD: 0.5% ± 0.08 KS: 4% ± 0.8	Low PD-1 expression overall which could be explained by subjective nature of flow cytometry gating.
Pesce et al. (2017)	Human	CMV+/CMV- healthy adults	PD-1	NK PD-1 0–10%, higher in CMV+	Links NK PD-1 to CMV+ serostatus, although 25% of PD-1- donors were CMV+.
Hsu et al. (2018)	Mouse	Intra-tumoral	PD-1	^b^NK PD-1 0-70%	1st study linking anti-tumor effects of anti-PD-1 therapy to direct NK cell effects. PD-1 expression on NK cells only observed in tumors, and expression levels highly variable across and within tumors.
Lieberman et al. (2018)	Human	Healthy donors	PD-1	Pre-activation ~5% Post-activation ~50%	PD-1 on more functional NK cells, not exhausted NK cells and only after 12 days of maximal activation.
Quatrini et al. (2018)	Mouse	MCMV infection	PD-1	MFI: MCMV>UI (spleen only)	1st paper linking tissue specific expression of PD-1 on NK cells to glucocorticoids and neurohormonal axis. Unlike Hsu paper, PD-1 expression only observed on splenic NK cells.
Zhang et al. (2018)	Human Mouse	Intra-tumoral	TIGIT PD-1	^c^Mouse tumor- infiltrating NK:TIGIT: 50–80%PD-1: <10%	1st paper showing influence of TIGIT on intra-tumoral NK cell function. Unlike Hsu paper, PD-1 expression on intra-tumoral NK cells consistently <10% across cell lines (CT26, B16, 4T1).
Alvarez et al. (2019)	Mouse	MCMV infection Cytokine treated	PD-1 TIGIT Tim-3	PD-1 ^d^Control: <10% Acute: <10% Chronic: <10% TIGIT Control: ~10% Acute: 25–40% Chronic: 10–25% Tim-3 Control: <10% Acute: 15–30% Chronic: <10%	PD-1 low on NK cells from control and stimulated mice. TIGIT and Tim-3 correlate with activation, but not maintained with chronic stimulation. Suggests NKG2D important in exhaustion phenotype.

a *PD-1 therapeutic antibody used in study later found to bind receptor distinct from PD-1*.

b *Variable across tumor cell lines*.

c *Human intra-tumoral NK cell TIGIT expression 10-80%*.

d *Untreated mice*.

*PTLD, post-transplant lymphoproliferative disease; RCC, renal cell carcinoma; CMV, cytomegalovirus; MCMV, murine cytomegalovirus; TILs, tumor infiltrating lymphocytes; MFI, mean fluorescence intensity*.

Regardless of the mechanism by which dysfunction has occurred, there must be a decrease in some effector function to label a cell as “dysfunctional.” For NK cells, this includes reductions in the expression of IFNγ (as shown either by intracellular staining by flow cytometry or by ELISA detection of secreted IFNγ), granzyme B, TNFα, CD107a (marker of degranulation), antibody dependent cell-mediated cytotoxicity (ADCC) via CD16, or decreased target cell cytotoxicity assays. In addition to decreased effector function, there should also be a concordant, subset specific increase in an exhaustion-associated marker (PD-1, TIGIT, TIM-3, LAG-3, etc.). Some of the more prominent studies examining NK cell exhaustion have focused of cancer patients including NK cells from the tumor microenvironment (Benson et al., 2010; da Silva et al., 2014; MacFarlane et al., 2014; Beldi-Ferchiou et al., 2016; Seo et al., 2017; Vari et al., 2018; Zhang et al., 2018; Sun et al., 2019) and in the context of chronic viral infections or prolonged pro-inflammatory cytokines (Wiesmayr et al., 2012; Felices et al., 2018; Alvarez et al., 2019; Zhang et al., 2019). Common to these reports is the identification of decreased NK effector function to first identify NK cells as exhausted–most notably IFNγ by flow cytometry detection. These cells are then shown to have increased expression of an established exhaustion-associated marker.

As PD-1 is the prototypical T-cell exhaustion marker, many studies have identified an association of PD-1 expression on NK cells with exhaustion. However, expression of PD-1 by NK cells remains controversial, and other studies have demonstrated TIGIT and to a lesser extent TIM-3 and LAG-3 to be more critical NK exhaustion markers. One of the early studies investigating PD-1 expression on NK cells was in pediatric post-transplant lymphoproliferative disease (PTLD) (Wiesmayr et al., 2012). This study noted that peripheral NK cells in patients with PTLD were phenotypically and functionally distinct from healthy donors or patients with asymptomatic EBV viremia. In these PTLD patients, there was decreased NKp46 and NKG2D expression, while PD-1 expression was increased compared to healthy controls and asymptomatic EBV carriers. The authors also showed functional differences between NK cells derived from PTLD patients compared to healthy controls (decreased CD107a, IFNγ), with augmentation of NK function in PTLD patients when they were treated with anti-PD-1 *in vitro*. However, the authors did not assess for differences in PD-1+ vs. PD-1- NK subsets, which is important since one would expect PD-1 negative NK cells in PTLD patients to function similarly to those of healthy donors. More so, evaluating the effects of anti-PD-1 therapy on PD-1+ and PD-1- subsets would be important to confirm the specificity of these findings.

Another important issue that the study by Wiesmayr et al., and other similar studies highlight is the challenge of determining an NK population to be “positive” when flow cytometric gating is used and does not delineate distinct populations. This can lead to wide variability in reported expression because small changes in flow cytometry gating dramatically alter the percent positive population. Additional studies in human cancer patients (Benson et al., 2010; Vari et al., 2018) have also shown PD-1 expression on NK cells, although interpretation of these studies may be limited by a wide variation in PD-1 expression in healthy controls (Vari et al., 2018) and a later finding that a novel antibody putatively identifying PD-1 on NK cells in multiple myeloma patients (Benson et al., 2010) appears to bind a separate receptor rather than PD-1 (Miller and Lanier, 2019).

Using murine models, Hsu et al., identified PD-1 expression on intra-tumoral NK cells, although these studies did not correlate PD-1 expression on NK cells with dysfunction (Hsu et al., 2018). The authors did observe significant variability in PD-1 expression across tumors (and even within replicates of the same tumors). For example, in RMA-S tumors (MHC-I negative lymphoma) PD-1 expression reached as high as 70%, while in RMA tumors (MHC-I positive lymphoma) expression varied between 20–50%, 0–60% in CT26 (colon cancer), 0–20% in B16 (melanoma), <5% in C1498 (AML), and 0–25% in 4T1 (breast). Hsu et al., also demonstrated significant anti-tumor effects when checkpoint blockade therapy targeting PD-1/PD-L1 pathway was administered *in vivo*. Although Hsu et al. do not directly address whether PD-1+ NK cells represent an “exhausted” subset and no comparison of the functional capabilities between PD-1+ vs. PD-1- intra-tumoral NK cells is shown, an earlier report from this group using similar mouse tumor models classified intra-tumoral NK cells as “anergic” (Ardolino et al., 2014) based on decreased expression of CD107a and IFNγ pre- and post-cytokine stimulation *in vivo*. Importantly, the authors did not observe NK cell anergy in MHC-I expressing tumors, suggesting that active inhibitory signaling via MHC-I and Ly49 receptors prevents exhaustion through tempering or tuning of activating signals. Interestingly, although PD-1 expression was variable but detectable by flow cytometry on intra-tumoral NK cells in this analysis (Hsu et al., 2018), there was no evidence of PD-1 expression on splenic NK cells, suggesting tissue specific effects. Moreover, these results are in contrast to a separate report from Quatrini et al., showing PD-1 expression restricted to splenic NK cells during MCMV infection, with no evidence of PD-1 expression from NK cells isolated from other organs or tissues. Importantly, this study uncovered a link between glucocorticoid signaling and the immune response to infection by NK cells. Although the authors did not directly link PD-1 expression to NK dysfunction, their findings of increased immunopathology in the spleens of mice with NK-specific PD-1 gene deletion suggested a novel role for NK cells in the neurohormonal response to infection and a physiological adaptation of NK dysfunction (Quatrini et al., 2018).

Other studies have examined exhaustion marker expression on NK cells in murine models and human cancer patients and observed different results regarding the key mediators of this process. For example, Zhang et al., examined intra-tumoral NK cells from multiple subcutaneous mouse tumor models and identified the co-inhibitory receptor TIGIT (T cell immunoglobulin and ITIM domain) as the critical marker for dysfunctional NK cells (Zhang et al., 2018). In this and other papers, PD-1 expression was minimal on NK cells, including intra-tumoral NK cells. In fact, TIGIT+ NK cells displayed decreased IFNγ, TNF, CD107a, and TRAIL expression consistent with decreased effector function. TIGIT blockade reversed NK dysfunction with superior anti-tumor effects in multiple murine tumor models.

One of the initial descriptions of TIGIT highlighted the ubiquity of TIGIT expression on healthy NK cells and its ability to bind ligands PVR (CD155) and PVRL2 (CD112) (Stanietsky et al., 2009). In this study TIGIT ligation was associated with decreased NK cytotoxicity. These ligands can be expressed on tumor cells and elicit an inhibitory signal to tumor-infiltrating NK cells (Sanchez-Correa et al., 2019). TIGIT+ NK cells may also be inhibited within the tumor microenvironment by MDSCs expressing the cognate ligands (Sarhan et al., 2016), thus making TIGIT a potentially prominent inhibitory receptor through various mechanisms. Earlier work on TIGIT and T cells showed that TIGIT expression did not have cell-intrinsic effects (Yu et al., 2009), but was based on CD226 interactions and ligand binding (Johnston et al., 2014). It has not been definitively determined if TIGIT expression marks intrinsically dysfunctional NK cells or if ligand binding is required for inhibitory effects, however expression of the inhibitory receptor TIGIT appears prominent both on human and mouse NK cells with potentially important clinical benefits.

Other investigators examining NK cell exhaustion have identified other markers of exhausted NK cells distinct from PD-1 and TIGIT. For example, a study in melanoma patients found a correlation between peripheral NK expression of TIM-3 and disease stage (da Silva et al., 2014), suggesting cancer progression and greater burden of disease induced NK exhaustion via increasing TIM-3 expression. Notably, these authors observed limited to no expression of PD-1 on NK cells (≤2%) and found no difference in PD-1 expression between melanoma patients and healthy donors. A more recent study utilizing CyTOF to analyze infiltrating immune cells in non-small cell lung cancer patients similarly detected virtually no PD-1 expression on intra-tumoral NK cells from 20 separate donors (Datar et al., 2019). Taken together, these studies highlight discrepancies in the evidence for and against exhaustion marker expression in NK cells, including PD-1. In addition, since these studies focus on cell surface marker expression (since this is usually viewed as mechanism to target NK cells therapeutically), there is less in-depth assessment of how expression of these markers is contributing to NK function or dysfunction and whether expression of these markers is adaptive, maladaptive, or potentially both.

Indeed, some studies have examined how stimulation of NK cells can both augment function and induce dysfunction, often simultaneously. For example, Alvarez et al. (2019) evaluated the occurrence of NK cell exhaustion after chronic stimulation (using viral and cytokine models) and proposed a paradigm of NK cell exhaustion similar to T cell/LCMV exhaustion. Specifically, the authors noted that prolonged cytokine exposure with IL-15 for >5 days or following MCMV infection stimulated sustained NK cell proliferation which then lead to decreased Ki67, IFNγ, and granzyme B expression. These exhausted NK cells were characterized by increased expression of KLRG1, decreased expression of cytotoxicity trigger NKG2D, and decreased expression of transcription factor Eomes. In keeping with discrepancies in identifying consistent markers of NK exhaustion, Alvarez et al., detected <5% expression of PD-1 on murine NK cells. Similarly, the finding of decreased expression of Eomes on exhausted NK cells was also discrepant to the classic phenotype of exhausted CD8+ T cells where Eomes expression is increased (Buggert et al., 2014).

Cytokine-based induction of NK dysfunction was also investigated by Felices et al. (2018) who detected differences in human NK cell responses when exposed to either continuous or intermittent IL-15. Though the authors did not define a clear phenotype of exhausted NK cells, they did identify significant differences in expression of CD107a, IFNγ, and NK cytotoxicity. The authors also showed that continuous IL-15 increased NK proliferation and decreased survival, secondary to alterations in NK cell metabolism induced by cytokine exposure which were partially mitigated by mTOR inhibition. While continuous IL-15 exposure appeared to induce an exhausted state in the work by Felices et al., combinations of IL-12, IL-15, and IL-18 were able to generate cytokine-induced memory NK cells (Cooper et al., 2009; Romee et al., 2012). It is currently unclear how the different cytokines act in concert to generate a functionally improved NK cell, while acting individually they appear to induce dysfunction. This suggests significant context-dependent effects from cytokine exposure as well as a narrow window between augmentation of function and induction of dysfunction.

Notably, IL-15, in addition to other cytokines (Cooper et al., 2009; Romee et al., 2012), also has been linked to the generation of memory-like NK cells and is necessary for the *de novo* generation of NK cells (Caligiuri, 2008). However, key papers have also highlighted the detrimental effects of IL-15 on NK cell malignant degeneration in the context of prolonged pro-inflammatory exposure. For example, Fehniger et al., showed that IL-15 transgenic mice develop fatal leukemia from NK and/or CD8 T cell infiltration (Fehniger et al., 2001) and *in vitro* studies from the same group observed that LGL leukemia can be induced from prolonged culture of human NK cells with IL-15, though this was on the order of >6 months exposure (Mishra et al., 2012). While neither of these studies investigated NK exhaustion or dysfunction in these contexts, they do provide evidence for the detrimental effects of prolonged cytokine exposure and the resulting dysfunction that can be attributed to exhaustion when examined under the appropriate lens.

Similar to the paradigm that T cell PD-1 expression can represent both early activation and exhaustion following prolonged antigen exposure depending on the kinetics of TCR engagement (Ahn et al., 2018), it has also been proposed that PD-1 expression on NK cells may delineate the most activated NK cells following *in vitro* stimulation (Lieberman et al., 2018). In these experiments, human NK cells were expanded using membrane bound-IL-15 and 4-1BBL transfected K562 cells supplemented with rhIL-2 and shown to upregulate PD-1. The PD-1+ NK subset (~30–70% by day 12, regardless of CD56^bright^ or CD56^dim^ expression) was also shown to have increased expression of the activation marker CD69. Along with the increase in PD-1 expression seen over 12 days of *in vitro* culture, these authors also observed decreased Tbet expression and increased Eomes expression on NK cells as shown by RNA analysis (although PD-1 mRNA was not assessed). Importantly, the interpretation of flow cytometric results such as those by Lierberman et al., using median fluorescence intensity (MFI) assessed at different time points is difficult. Unlike using the endpoint of percent positive cells (for marker expression) compared to control populations using fluorescence minus one (FMO) or isotype antibodies, the use of MFI as a readout of marker expression is susceptible to changes in light scattering properties across conditions and reagents (Vitale et al., 1989; Zamai et al., 1998). As a result, comparing MFI across time and experiments can introduce error as differences in treatment, internal controls and/or flow cytometric parameters may significantly alter the baselines and variance among samples. This is especially true as NK cells increase in size and granularity with activation (Zarcone et al., 1987).

Though controversies exist in defining exhausted NK cells and identifying exhaustion-specific markers on NK cells, there is a lack of investigation into the beneficial role of NK exhaustion. With limited evidence for direct NK-mediated autoimmunity, it is more likely that upregulation of exhaustion markers may serve to limit toxicity under certain highly activating conditions or limit immunopathological effects mediated by other cell types. This has been recently examined in the context of viral hepatitis and autoimmune cholangitis. In the viral hepatitis study, it was determined that liver resident NK cells express PD-L1 to bind and inhibit T cells (via PD-1) to limit immunopathology from anti-viral T cells, while leading to decreased viral clearance and persistence (Zhou et al., 2019). Similarly, a study investigating autoimmune cholangitis showed that liver resident NK cells inhibit CD4 T cells to limit the severity of autoimmune cholangitis, and loss of liver resident NK cells worsens the disease (Zhao et al., 2019). Distinct from immunopathology, however, PD-L1 has also been shown to be induced on NK cells by tumors and augmentable by anti-PD-L1 therapies for an anti-tumor effect (Dong et al., 2019). While only PD-L1 on NK cells has been implicated in these studies, it is probable that other exhaustion markers may be upregulated on NK cells to limit NK-specific toxicities, or to indirectly limit T cell-mediated immunopathology.

## Other Dysfunctional States

In recent years immune exhaustion has received the most attention due to the ability to identify exhausted T cells (based primarily on PD-1 expression) and the ability to antagonize PD-1 signaling using therapeutic antibodies with well-publicized clinical benefits. However, other states of cellular dysfunction have also been described and characterized, notably anergy and senescence, as well as deprivation and suppression, which are critical to immune cell function.

## Anergy

In the broadest sense, T-cell anergy represents a state of intrinsic functional inactivation (Schwartz, 2003). Schwartz (who provided some of the initial descriptions of T-cell anergy) divided the term into two categories—clonal anergy and adaptive tolerance. Both states are characterized by decreased proliferation and decreased IL-2 production, but clonal anergy derives from insufficient activation and appears to occur in mature T cells, while adaptive tolerance occurs secondary to insufficient co-stimulation of naïve T cells (Schwartz, 2003; Chiodetti et al., 2006). Further evaluation of these anergic states identified distinct biochemical pathways associated with clonal anergy and adaptive tolerance, respectively (Chiodetti et al., 2006). Despite these differences, it is critical to understand that both states appear to have evolved as a tolerance mechanism aimed at limiting autoimmunity, thus attempts to reverse anergy (as has been pursued for exhaustion) are liable to prove detrimental while potentially also yielding minimal benefit.

Should NK cells follow the same paradigm set forth by T cells, then anergy may occur following an insufficient activating signal (adaptive tolerance) or following a strong stimulus without adequate co-stimulation (clonal anergy). A sequential signaling model for NK cells analogous to naïve T-cell activation was recently proposed by Vidard et al., Here, the authors described a three-signal sequence required for maximal NK cell activation and proliferation (Vidard et al., 2019). The authors showed that maximal NK proliferation required NK-aAPC contact, CD137 (4-1BB) activation, and cytokine (IL-2, IL-15, IL-21) signaling. Once removed from maximally activating conditions, cytokine alone was capable of maintaining cytotoxic function against target cells as evidenced both by target cell lysis and NK cell IFNγ production (Vidard et al., 2019). Hypothesizing a similar mechanism to T cells, NK cells would thus become “anergic” if CD137 signaling is absent at the time of NK-aAPC contact, which may be the case when NK cells are within the tumor microenvironment where NK cells contact MHC-I negative tumor cells and may receive IL-2 and/or IL-15 cytokine signaling, without ligation of CD137, which is typically provided by antigen presenting cells. This sequence of events is supported by Ardolino et al., who showed reversal of NK cell anergy on intra-tumoral NK cells when exogenous IL-12 and IL-18 was given systemically (Ardolino et al., 2014). Overall, although these data support the concept of NK cell anergy via inadequate 3-signal activation, it is difficult to differentiate reversal of NK anergy in these models from *de novo* activation of resting NK cells by cytokine exposure, a process that has been well-established for the generation of lymphokine activated killer (LAK) cells (activated NK and CD8 T cells) and successfully used in clinical trials (Rosenberg, 1985, 2014; Rosenberg et al., 1985, 1987) as well as for generation of cytokine-induced memory NK cells, as discussed above.

Also comparable to T cell anergy (Otten and Germain, 1991), other groups have identified “split anergy” in NK cells (characterized by simultaneous loss of one specific function, with corresponding gain of a separate effector function) (Jewett and Bonavida, 1996; Jewett et al., 1996, 1997, 2006; Tseng et al., 2015). As per the extensive work by Jewett and Bonavida, NK cell “split anergy” occurs following ligation of CD16 and is typically characterized by the subsequent loss of CD16 expression with gain of cytokine secretion abilities. The beneficial effects of NK split anergy appear to be increased control of cancer stem cells (CSCs) through induction of target cell differentiation (Tseng et al., 2014, 2015), which is consistent with other studies that have observed NK cell targeting of CSCs (Ames et al., 2015; Luna et al., 2017).

The advent of cytokine therapy and other monoclonal-based immunotherapeutics for cancer has created a situation for T cells that may have otherwise never been encountered in nature—that is out of sequence signal 3. This novel scenario and its downstream consequences were previously investigated by multiple labs independently and a novel, anergy-like T-cell dysfunction was identified (Urban and Welsh, 2014; Sckisel et al., 2015). To date, these adverse effects have not been identified in NK cells, and pro-inflammatory cytokines have consistently been shown to enhance NK effector function and proliferation (Biron et al., 1999), though not to a maximal effect as illustrated by Vidard et al. (2019). However, key aspects of anergy have been observed/invoked in the context of licensing as NK cells develop additional immunoregulatory mechanisms to balance appropriate target recognition with tolerance. In this context, these features of anergy likely have host benefits in the context of NK cell maturation and function and highlight another key difference between NK cells and T cells as resting NK cells can kill target cells provided appropriate positive signals are provided and steady-state inhibitory signals are removed (Orr and Lanier, 2010). Interestingly, in studies investigating the topic of NK tolerance, cytokines previously described in NK memory and anergy studies (such IL-12 and IL-18) were also capable of restoring NK function in unlicensed NK cells (Orr and Lanier, 2010), and unlicensed (tolerant, hyporesponsive) NK cells were shown in a related study to predominate in the NK response to MCMV (Orr et al., 2010). These findings regarding overlap between NK tolerance and anergy add complexity to our understanding of whether NK dysfunction is helpful or harmful to the host.

## Senescence

The universal phenomenon of replicative senescence (and cellular aging) appears related to shortening of telomeres, the eventual recognition of genomic DNA as double-strand breaks, the implementation of repair machinery, and the ultimate arrest of the cell cycle to halt replication and prevent compounding of genomic instability (Campisi, 2013). Senescent T cells are observed in both aged humans and after prolonged *in vitro* culture (Spaulding et al., 1999), and they are phenotypically characterized by decreased CD28 expression (Effros et al., 1994). Functionally, senescent T cells have decreased replicative ability (Spaulding et al., 1999), are associated with increased production of pro-inflammatory cytokines TNFα and IL-1 (Dayan et al., 2000), several proteases (Callender et al., 2018), and reduced apoptosis (Spaulding et al., 1999). In contrast to other forms of immune cell dysfunction, senescence appears to be a universal byproduct of prolific replication and not a direct result of antigen-specific stimulation or other stimulatory or inhibitory conditions. Characterization of NK senescence has not been specifically elucidated, but given the universality of cellular senescence, it is presumed that senescent NK cells are also associated with a pro-inflammatory senescence-associated secretory phenotype (SASP) and decreased proliferative capacity. As senescence results from continued proliferation, techniques to generate high numbers of NK cells for therapy may induce senescence and promote dysfunction.

Understanding the lifespan of the cell is critical to gaining an understanding of NK senescence, though this is challenging as the lifespan of an NK cell is not clearly defined. Estimates of the *in vivo* half-life of murine NK cells are ~7–10 days (Yokoyama et al., 2004), and possibly <10 days in humans (Nayar et al., 2015), though this view has been expanded by advances in cell identification and barcoding techniques in non-human primates showing unique developmental pathways of NK subsets (Wu et al., 2014) and prolonged persistence (months) of specific NK clones (Wu et al., 2018). *In vitro*, this can be markedly manipulated, but the limit of healthy, normal human NK cells to grow in culture appears to be ~15 weeks (Fujisaki et al., 2009a). In mice, *in vitro* proliferation of healthy NK cells is ~7–10 days before apoptosis-associated changes occur, though NK cells derived from *P53* knockout mice have been cultured for over 1 year under specific conditions (Karlhofer et al., 1995). Fujisaki et al. (2009a) augmented this limit by culturing healthy human NK cells in the presence of the transfected K562 cell line bearing membrane bound 4-1BBL and IL-15, supplemented with 10 IU/mL IL-2. Using this system, healthy NK cells were able to undergo 15 weeks of culturing and 20 population doublings before losing replicative ability and dying despite continued stimulation. When NK cells were transfected with the *TERT* gene, replicative ability was restored, and cells could now be cultured almost 160 weeks with continued cytotoxicity. Transfected NK cells, however, were not able to grow autonomously in NSG mice and still eventually developed senescence *in vitro*, though at a much later time point compared to the non-transfected population (Fujisaki et al., 2009a). Additional studies using similar transfected K562 cells with membrane bound IL-21 (rather than IL-15) showed superior expansion of human NK cells compared to membrane bound IL-15 K562 cells (Denman et al., 2012). NK cells expanded with the IL-21-bearing K562 cell line exhibited increased telomere length which may be related to differences in STAT signaling and *TERT* regulation. These studies highlight that under ideal, highly activating and stimulatory conditions, healthy human NK cells are most limited by senescence at much later time points than believed to occur *in vivo*, but not by other mechanisms of dysfunction (i.e., exhaustion). The reason for this is unclear as prior reports would suggest that continuous cytokine exposure can induce NK cell exhaustion. It is critical to note that *ex vivo* expansion using the transfected K562 cell line utilizes membrane bound ligands, particularly 4-1BBL, which is not present in other studies utilizing exogenous cytokine alone. Although membrane-bound ligands may be more physiologic, it does not reflect the clinical usage of exogenous cytokine (IL-2 or IL-15) that may induce distinct immunologic effects. Perhaps these supraphysiologic conditions overcome any inhibitory mechanisms NK cells attempt to implement or alternatively there are no ligands present to bind and send inhibitory signals to the NK cells.

The phenotype of senescent NK cells has also been investigated using a similar *in vitro* expansion system based on transfected K562 cells bearing membrane bound 4-1BBL and IL-21 supplemented with 100 U/mL IL-2 (Streltsova et al., 2018). These authors noted that following prolonged culture for 2–8 weeks, NK cell expression of inhibitory receptor NKG2A was increased and maturation marker CD57 was lost. However, there was no difference in IFNγ production or NK cell cytotoxicity against target cells between CD57 positive and negative subsets, and it was unclear if there was a proliferation difference between the CD57 subsets, which would be a hallmark of senescent cells. In prior work evaluating the CD57 subsets, Lopez-Verges et al., identified critical differences between these cell populations when differentiating the mature CD3-CD56^dim^ NK cells between CD57+ and CD57- (Lopez-Vergès et al., 2010). The authors found that CD57+ NK cells proliferated less when exposed to either cytokine or target cells, although the CD57+ NK cells had greater expression of IFNγ and greater cytotoxicity with CD16 stimulation. The authors also observed that IL-2 induced CD57 expression on ~30% of NK cells after 5 days in culture. With the co-culture NK expansion system (transfected K562-mbIL15-41BBL feeder cells with IL-2), they also showed that the CD57- subset exhibited increased proliferation compared to the CD57+ NK cells (Lopez-Vergès et al., 2010). These findings suggest that over the course of extended *ex vivo* NK expansion the more proliferative CD57- NK cells will disproportionately expand and outcompete the CD57+ population and result in a CD57- NK cell product. Given these results, a definitive phenotype of NK senescence has not been achieved and functional consequences of NK senescence are not clearly established.

The *in vivo* implications of aging on NK-cell senescence and resultant function has also been reviewed (Hazeldine and Lord, 2013) with hypothesized consequences such as increased viral infections, decreased anti-microbial functions and increased malignancies. Gounder et al., showed in healthy humans an age-dependent loss of total peripheral lymphocytes, but relative increases in the NK cell compartment, suggesting that NK cells were less susceptible to the effects of aging, particularly senescence. However, NK cells from younger donors expanded to a greater extent than those from older donors using IL-2-based *in vitro* stimulation, highlighting that NK cells were still susceptible to age-related dysfunction (Gounder et al., 2018). Murine studies have also shown age-related differences in NK cells (Beli et al., 2014; Nair et al., 2015), with one study showing decreased total and mature NK cells in the peripheral tissues in older mice aged 15–18 months, and an accumulation of mature NK cells within the bone marrow of these aged mice compared to younger mice aged 6–8 weeks (Beli et al., 2014).

Separate from their well-established anti-viral and anti-tumor effects, NK cells are also prominent within the gravid uterus early in gestation (Moffett-King, 2002) and senescence may have a beneficial role. These unique uterine NK cells have a proposed role in remodeling uterine vasculature to augment fetal circulation (Rajagopalan and Long, 2012; Rätsep et al., 2015). There is also evidence that this effect is mediated by persistent signaling and induction of a senescent phenotype in NK cells (Rajagopalan and Long, 2012). In their study, Rajagopalan and Long showed that persistent NK cell signaling via CD158d interactions with HLA-G induced a DNA damage response pathway which lead to senescent NK cells with a senescence-associated secretory phenotype and resultant effects on vascular remodeling (Rajagopalan and Long, 2012). This contrasts with the hypothesis that senescence is strictly the end-result of cellular replication and suggests that the induction of a senescent phenotype may be intentional and programmed in specific situations, such as providing marked benefit to the host and fetus during gestation.

## Deprivation, Inactivation, and Suppression

The essential role for cytokines in the survival and maintenance of NK cells has been well-established (Carson et al., 1997; Lindemann et al., 2003). Moreover, withdrawal of cytokine IL-15 has been shown to lead to rapid NK cell apoptosis within 24 h of withdrawal (Huntington et al., 2007). For NK cells activated and expanded using the co-culture system (feeder cells with cytokine typically IL-2 at 100 IU/mL), it seems likely that NK cells undergo rapid apoptosis following adoptive cell transfer into a host that does not provide these robust activating signals *in vivo* which occur *in vitro*. These phenomena are similar to cytokine deprivation which has been proposed to be the critical mechanism for regulating T cell contraction following effector T cell expansion (Vella et al., 1998; Strasser and Pellegrini, 2004; Yajima et al., 2006; Fischer et al., 2008). Though this hypothesis has been challenged (Prlic and Bevan, 2008), it clearly highlights the critical role for cytokine deprivation in shaping immune cell compartments. As NK cells are also intensely dependent on cytokines for survival, proliferation, and activation signals, it is likely that cytokine deprivation plays a major role in regulating NK cells and may be responsible for the underwhelming results of clinical trials using *ex vivo* activated and expanded NK cells.

In particular, clinical trials with *ex vivo* expanded NK cells have shown limited NK engraftment following adoptive transfer. For example, in a phase II trial using IL-2 activated allogeneic NK cells transferred into lymphodepleted recipients with recurrent ovarian or breast malignancies (*n* = 20), post-transfer subcutaneous IL-2 administration (10^7^ units, 3×/week for 2 weeks) led to *in vivo* NK expansion in only a single patient (expansion defined as ≥100 donor NK cells/μL whole blood at day 14). In this patient, adoptively transferred NK cells reached 40% of the lymphocyte pool by day 7 post-transfer, but then decreased to 4% of the lymphocyte pool by day 14 (Geller et al., 2011). Similar results regarding loss of donor NK cells were observed in a more recent study in AML patients (Romee et al., 2016). Allogeneic, haploidentical NK cells were activated *ex vivo* with IL-12, IL-15 and IL-18 then transferred into patients with AML. Patients received subcutaneous IL-2 (10^6^ IU/m^2^ every other day for six doses), and a similar loss of donor NK cells was observed, with most recipients having undetectable donor NK cells by days 14–21 post-transfer. The duration of NK engraftment appear even more challenging for NK-92-based clinical trials as reported from a small trial for hematologic malignancies (Williams et al., 2017). Of the patients who were evaluated for post-transfer NK-92 detection, most transferred cells were lost after 15 min, and circulating NK-92 cells were undetectable by 6 h post-transfer. These trials underscore the common challenge of maintaining NK cell engraftment and persistence following adoptive cell transfer of pre-activated or expanded NK cells that appear dependent on cytokines. Future directions for limiting the *in vivo* dependence of NK cells of supraphysiologic levels of cytokine may include methods to “wean” activated NK cells from cytokine prior to transfer, inhibitory blockade *in vivo* or genetically engineering expanded NK cells to make IL-15 or other stimulatory cytokines.

Inactivation of NK cells in specific contexts can alter functional readouts that are used for identification of dysfunctional states. This can be the case for the activating immunoreceptor NKG2D, a key mediator of NK-cell target recognition (Raulet, 2003). Initially shown on NK and T cells to be activated through stress-inducible MICA (Bauer et al., 1999), NKG2D recognizes MHC-I-related ligands that are rarely expressed on normal cells, but upregulated on “stressed” cells, resulting from viral infections or transformation (Raulet, 2003). These ligands, which are present in both human and mouse, are also able to induce functional changes on the NK cell upon NKG2D binding. When expressed on stressed cells, these ligands act as a target for NK cell killing, but these ligands have also shown to be shed from tumors to act as a decoy for NKG2D-targeting. These shed NKG2D ligands have been shown to negatively affect the effector function of both T cells (Groh et al., 2002) and NK cells (Song et al., 2006). In the work by Song et al., the authors detected soluble NKG2D ligands from gastric cancer cell lines that caused loss of NKG2D expression on NK cells, leading to a decrease in NK target cell lysis in cytotoxicity assays (Song et al., 2006). The influence of soluble NKG2D ligands has been complicated by further work showing an opposite effect in knockout and transgenic mouse models (Deng et al., 2015). In their work, Deng et al., found that shed MULT1 increased NKG2D expression on NK cells and increased effector function resulting in *in vivo* rejection of tumor. These results complicate interpretation of intra-tumoral NK cell function as these shed NKG2D ligands clearly alter cytotoxic capability, a critical readout for NK cell function.

Lastly, another mechanism which mediates NK dysfunction is by extrinsic suppressive signals that NK cells may be exposed to, particularly in the tumor microenvironment. The effects of TGF-β on NK suppression has been extensively described (Batlle and Massagué, 2019) and is one of the most well-established NK inhibitory cytokine/growth factors in the tumor microenvironment (Bellone et al., 1995; Bergmann et al., 1995; Zaiatz-Bittencourt et al., 2018). In fact, inhibition of NK cells by TGF-β has been shown in multiple pre-clinical models (mouse and human, *in vitro* and *in vivo*) (Li et al., 2009; Zaiatz-Bittencourt et al., 2018). These effects may greatly confound data examining intra-tumoral NK cell dysfunction as the extensive direct and indirect effects of TGF-β may be difficult to control for. Additionally, the source of TGF-β can be diverse (tumor, Tregs, macrophages, MDSCs, etc.) and the heterogeneity of infiltrating immune cells could proportionately alter TGF-β concentrations within tumor models and increase NK cell dysfunction. Additional work has shown the impact of MDSCs on NK cell function (Sarhan et al., 2016). Specifically, this group showed that the memory-like adaptive human NK cells express lower TIGIT and are thus less susceptible to MDSC-mediated inhibition through CD155/TIGIT binding. These data highlight the diverse mechanisms of NK dysfunction in the tumor microenvironment and the interplay between suppressive cells, inhibitory receptors, and NK effector function, and the challenges in identifying the etiology of NK dysfunction.

## NK Cells in Adaptive Immunity

While the classical definition of NK cells as non-T, non-B lymphoid cells of the innate immune system (Vivier et al., 2011) remains true, it has become increasingly evident that NK cells also possess specific traits of the adaptive immune system such as antigen specificity and memory recall (Sun et al., 2009; Vivier et al., 2011; Cerwenka and Lanier, 2016). The capacity of NK cells to recognize specific antigens (e.g., m157 in murine CMV) and display enhanced function when specific antigens are re-encountered, suggest, circumstantially at least, that immune checkpoints may also be operative in NK cells in order to limit autoimmunity and immunopathology, as this is one of the key proposed evolutionary roles for T cell exhaustion. However, the well-defined sequential activation steps required for generation of effector and memory populations in T cells are unidirectional in nature (Smith-Garvin et al., 2009), and T cells require antigen presentation and priming to occur before they are capable of an effector response. In contrast, NK cells are capable of target cell killing from a baseline state, given the presence of adequate activating signals and lack of inhibition, and unlike T cells they can more easily alternate between resting and activated states. Thus, the plasticity and potential for bidirectionality among resting and activated states likely impacts the expression of checkpoint/inhibitory markers (Lanier, 2008), and since NK cells are shorter-lived immune cells that can oscillate between resting and activated states, the benefit of exhaustion marker upregulation on these cells seems less necessary for appropriate immuno-regulation.

## Memory NK Cells and Dysfunction

In addition, the lifespan of NK cells is critically important to the debate regarding NK exhaustion as only long-lived immune cells would theoretically be worth the evolutionary investment of synthesizing and upregulating exhaustion markers to regulate chronic immune stimulation. Compared to T cells, the lifespan of an NK cell is proposed to be much shorter, although this topic remains an area of significant debate due to differences between humans and mouse models. Fundamental advances in the understanding of NK cell biology have been generated from mouse models (Sungur and Murphy, 2013), and while these advances have improved understanding of human NK cells, there are critical species differences that limit the application of murine data to human application, opening the door for novel immunocompetent models to study NK biology (Park et al., 2016; Canter et al., 2017). One of the most critical differences between mouse and human NK cells concerns the lifespan of an NK cell and the putative, long-lived “memory” NK cell. Early evidence for a memory response by NK cells was put forth by O'Leary et al. (2006) who identified contact hypersensitivity to a hapten upon re-exposure 4 weeks after the initial sensitization. This “recall” response was maintained in T- and B-cell deficient mice (*Rag2*^−/−^) but lost in mice devoid of all lymphocytes (*Rag2*^−/−^*Il2rg*^−/−^), thereby implicating NK cells in the mechanism. This concept was further developed in studies evaluating the influence of NK cells on early anti-viral responses and post-vaccine re-exposure responses (Horowitz et al., 2010a,b, 2012). Initial data showed the importance of NK cells in responding to the early malaria infection (within 12–18 h), responses which were dependent on CD4-derived IL-2 (Horowitz et al., 2010b). This phenomenon was exploited in post-vaccine NK recall responses which were also dependent on IL-2 produced from antigen-specific memory CD4 T cells (Horowitz et al., 2010a). These data were corroborated in humans immunized against malaria (Horowitz et al., 2012). The concept of intrinsic memory NK cells was expanded in a seminal paper by Sun et al. (2009) who showed an antigen specific memory response of Ly49H+ NK cells after murine cytomegalovirus (MCMV) infection, including after adoptive transfer of MCMV-exposed Ly49H+ NK cells into MCMV non-exposed recipients. The authors demonstrated that these transferred NK cells remained in the recipient for up to 70 days and could respond better than naïve NK cells following anti-NK1.1 stimulation *in vitro* with increased IFNγ expression. Similarly, adoptive transfer of Ly49H+ memory NK cells into DAP12-deficient neonatal mice also produced improved survival among the recipient mice following MCMV infection *in vivo*. These observations from murine studies indicate that long-lived memory NK cells occur in specific settings, and these memory NK cells can exhibit superior effector function without evidence for dysfunction as might be suspected for a longer-lived immune effector cell. In fact, although studies addressing this question are relatively limited to date, there are currently no reports demonstrating increased susceptibility to dysfunctional states (exhaustion, anergy, or senescence) in murine memory NK cells.

The proposed human correlate to the mouse Ly49H+ memory NK cell is the “adaptive” NK cell characterized by NKG2C expression which is proposed to expand following human CMV exposure and recognize target cells via binding of HLA-E (Gumá et al., 2004; O'Sullivan et al., 2015). Work by Jeff Miller's group at U. of Minnesota has showed that allogeneic hematopoietic cell transplant (HCT) recipients who experience CMV reactivation have a preferential expansion of NKG2C+ NK cells, with increased effector functions of this subset (Foley et al., 2012b). An additional study showed upregulation of NKG2C expression following HCT into CMV seropositive recipients (Foley et al., 2012a). However, the importance of NKG2C as a marker of memory NK cells has been challenged by observations that humans who carry homozygous null mutations for NKG2C do not appear to have any demonstrable viral-specific consequences in their immune responses from loss of NKG2C expression, although compensatory mechanisms have been proposed (Liu et al., 2016). Given the interaction between NK cells and CMV, Pesce et al., examined NK cell phenotype among CMV seropositive and seronegative subjects. They showed that only NK cells from CMV seropositive individuals expressed PD-1 (~25% of cells) vs. 0% in seronegative subjects (Pesce et al., 2017). While the authors noted several phenotypic and functional differences between PD-1+ and PD-1- NK cells from healthy donors, it was notable in this study that 199 of 200 healthy donors had PD-1 expression ≤10%. Other groups have also examined NKG2C+ expression as a surrogate for long-lived adaptive NK cells. Merino et al., reported that NKG2C+ NK cells upregulate checkpoint receptors PD-1 and LAG-3 following prolonged NKG2C agonist activity (Merino et al., 2019). In their study, the authors showed that IL-15 combined with anti-NKG2C signaling led to LAG-3 upregulation, and the LAG-3+NKG2C+ NK cells were less functional as determined by decreased IFNγ expression and diminished cytotoxicity following co-culture with the erythroleukemia cell line and prototypical NK target, K562. Notably, these endpoints of dysfunction are the same features classically associated with T cell exhaustion. Also of note, in contrast to the results obtained by Pesce et al. (2017), Merino et al., detected no PD-1 expression on NKG2C+ or NKG2C- cells at baseline. And while anti-NKG2C and IL-15 led to increased PD-1 expression on the NKG2C+ NK cells only, it appears the NK2GC+ NK cells only represented ≤5% of the total NK cells in the *in vitro* assay (Merino et al., 2019).

Another important point is that the results from murine and human studies investigating memory/adaptive NK cells appear to show conflicting evidence regarding the extent of NK dysfunction and exhaustion. Data from mouse models suggests that memory NK cells exhibit superior responses compared to naïve NK cells as seen by increased IFNγ and improved survival with infection, suggesting a lack of dysfunction or exhaustion. In contrast, data from human studies suggest that adaptive NK cells are the principal NK cell subset susceptible to exhaustion as seen by increased PD-1 and LAG-3 expression and decreased functional effects. Ultimately, NKG2C+ NK cells appear to be a minority of peripheral NK cells and are lacking in humans with homozygous null mutations, with no apparent adverse effects. However, the therapeutic potential of expanded NKG2C+ NK cells remains to be determined, as CMV exposure is known to alter the KIR repertoire with long-lasting alterations in the inhibitory and activating KIR profile (Béziat et al., 2013).

Apart from antigen-induced memory NK cells, other recent studies suggest that human (Romee et al., 2012) and mouse (Cooper et al., 2009) memory NK cells can be induced solely by cytokine exposure, and that these cytokine-induced memory NK cells can be exploited to elicit meaningful anti-leukemia effects in cancer patients (Romee et al., 2016). Notably, however, although an NK “memory” phenotype is postulated as the mechanism of action in a clinical trial using cytokine-stimulated memory NK cells, this conclusion is limited by data showing that transferred NK cells following *ex vivo* cytokine expansion (and “memory” formation) rapidly disappear from the recipients' peripheral blood by day 7–14 post-transfer (Romee et al., 2016). While the concept of memory NK cell induction by cytokine signaling alone is intriguing and warrants further investigation, an examination of the significance of this subset of NK cells is hindered by observations showing that this NK cell subset does not persist beyond a maximum of 21 (Romee et al., 2012) or 22 (Cooper et al., 2009) days post-transfer. Although exhaustion or other manifestations of dysfunction may underlie their limited lifespan *in vivo* (as observed with other NK cellular therapies), a formal assessment for exhaustion or dysfunction parameters have not been performed.

## Conclusion

Characterizing and differentiating between anergy, exhaustion and senescence has led to critical discoveries in the biology of T cell dysfunction, most notably the reversal of exhaustion with blocking antibodies which can lead to dramatic clinical anti-tumor benefits. As the same paradigm is being applied to NK cells through application of checkpoint blockade therapy, including both PD-1 and PD-L1 inhibitors, it is critical to delineate when, how, and why anergy, exhaustion and senescence of NK cells occurs in order to better understand their complex biology and thus fully realize the potential of NK-based therapies.

## Author Contributions

SJ, WM, and RC conceptualized the manuscript, reviewed the literature, wrote, and edited the final paper.

### Conflict of Interest

The authors declare that the research was conducted in the absence of any commercial or financial relationships that could be construed as a potential conflict of interest.
